# The impact of assisted reproductive technology in twin pregnancies complicated by intrahepatic cholestasis: a five-year retrospective study

**DOI:** 10.1186/s12884-022-04610-5

**Published:** 2022-03-31

**Authors:** Qianwen Zhang, Yu Xu, Yunhui Gong, Xinghui Liu

**Affiliations:** 1 Department of Obstetrics and Gynecology, West China Second University Hospital, Sichuan University, Chengdu, Sichuan China; 2 Key Laboratory of Birth Defects and Related Diseases of Women and Children, Ministry of Education, Sichuan University, Chengdu, Sichuan China

**Keywords:** Intrahepatic cholestasis of pregnancy, Twin pregnancies, Ursodeoxycholic acid, Assisted reproductive technology, Spontaneous conception

## Abstract

**Background:**

Intrahepatic cholestasis of pregnancy is one of the common complications during pregnancy, and ursodeoxycholic acid has been recommended as the first-line drug. However, if the assisted reproductive technology may increase adverse perinatal outcomes of twin pregnancies complicated by intrahepatic cholestasis is disputed, we aimed to investigate perinatal outcomes between twin pregnancies by assisted reproductive technology versus spontaneous conception, based on these women accepted ursodeoxycholic acid treatment.

**Methods:**

From January 2014 to January 2019, we retrospectively analysed the clinical data of twin pregnant women with intrahepatic cholestasis, excluding those who did not receive ursodeoxycholic acid treatment. In total, 864 women were included, among whom 500 conceived by assisted reproductive technology and 364 conceived by spontaneous conception. The primary assessment for perinatal outcomes included premature birth, meconium-stained amniotic fluid, low Apgar score, neonatal intensive care unit and still birth, and secondary indicators were serum bile acid and liver enzymes level during medication, so we also finished subgroup analysis based on different elevated bile acid level and drug usage. The statistical analysis was performed by SPSS 22.0.

**Results:**

The study demonstrated that compared to spontaneous conception, assisted reproductive technology conceived twin pregnancies diagnosed as intrahepatic cholestasis earlier (*p* = 0.003), and lower birth weight (*p* = 0.001), less incidence of preterm delivery (*p* = 0.000) and neonatal intensive care unit admission (*p* = 0.001), but the rate of meconium-stained amniotic fluid, low Apgar score and still birth have no statistic differences. Moreover, the subgroup analysis showed no significant difference in elevated bile acid levels and medication between assisted reproductive technology and spontaneous conception groups.

**Conclusions:**

The assisted reproductive technology may increase the risk of early-onset intrahepatic cholestasis in twin pregnancies, but it does not seem to increase adverse effects on bile acid levels and perinatal outcomes. Regardless of ursodeoxycholic acid used alone or combination, the effect of bile acid reduction and improving perinatal outcomes in twin pregnancies is limited. Our conclusions still need more prospective randomized controlled studies to confirm.

## Background

Intrahepatic cholestasis of pregnancy (ICP) is clinically characterized as pruritus, elevated serum aminotransferase, and total bile acid level (TBA ≥ 10 μmol/L), and those symptoms can quickly resolve after delivery [1]. The etiology and pathogenesis of ICP are not fully understood, relevant factors may involve genetics, hormones, or the environment, pregnant women have advanced maternal age, preexisting hepatobiliary disease, previous history, and multiple gestations are at particular risk for cholestasis of pregnancy [2, 3]. In addition, TBA ≥ 40 μmol/L, gestational hypertension, preeclampsia, and other comorbidities are associated with adverse perinatal outcomes in ICP [4].

Compared with normal pregnancy, adverse perinatal outcomes may more likely occur in ICP, mainly including still birth, meconium-stained amniotic fluid (MSAF), neonatal respiratory distress syndrome (RDS), spontaneous and iatrogenic preterm birth [5–7]. The still birth rate beyond 37 weeks of gestation attributable to ICP is approximately 1.2%, so active perinatal management is crucial to prevent unpredictable still births [8]. Several drugs could be used for ICP, Ursodeoxycholic acid (UDCA) has been recommended as a first-line agent to reduce serum bile acid levels and relieve itching symptoms. UDCA has few side effects and is safe for the fetus, and S-adenosyl methionine (SAMe) could be an alternative or combination therapy [9–11]. For pregnant women who failed UDCA alone, someone researchers found rifampicin may produce a synergistic effect with UDCA, and could promote the conversion and excretion of bile acids, further improving liver function and reducing bile acid levels [12].

Even though medication helps improve laboratory abnormalities, it is still uncertain whether it could reduce adverse perinatal outcomes [13, 14], so someone begins to question aggressive UDCA usage for women with mild TBA levels [15, 16] or try to find more predictable indicators for adverse perinatal outcomes in ICP [11]. The varied incidence of ICP is 0.3%–5.6% among pregnant women, but it could be 20.9% ~ 24.6% during twin pregnancies because ART has been widely applied in infertile women [17–19]. Compared to spontaneous pregnancy, it has not been confirmed that if ART conceived twins may develop a higher incidence of adverse perinatal outcomes [20–22]. The present study was based on evaluating UDCA treatment by subgroup analysis and aimed to determine whether ART is associated with adverse outcomes during twin pregnancies with ICP.

## Methods

**Fig. 1:**
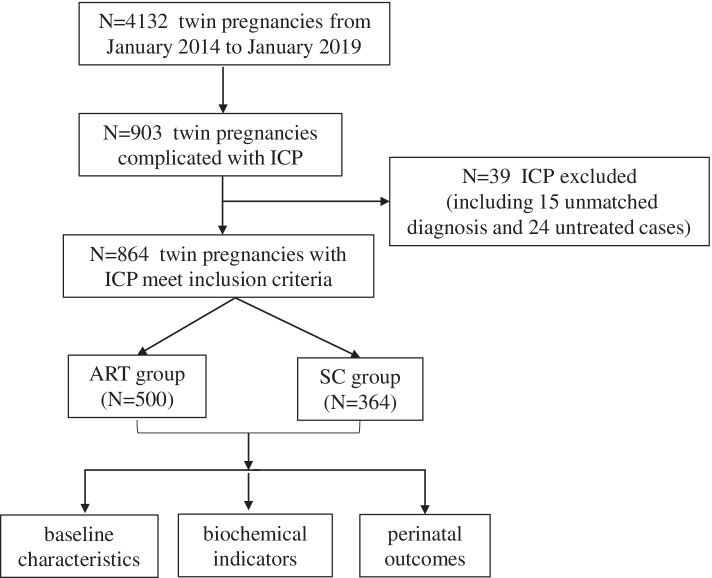
Flow chart shows detailed inclusion process

This retrospective cohort study included twin pregnancies diagnosed as ICP delivering after 28 gestational weeks, from January 1^st^, 2014 to January 31^st^, 2019. First, twin pregnancies with ICP were searched by the 10th International Classification of Diseases (ICD-10) code, and excluding pregnant women unmatched diagnosis or untreated, clinical data of all included cases were abstracted from the computerized medical records system. Every ICP patient was reconfirmed to have a fasting serum TBA level > 10 μmol/L, with or without pruritus and elevated aminotransferases, and no additional diseases that may cause similar symptoms or laboratory abnormalities. According to the Chinese Guidelines, twin pregnancies with mild serum TBA levels (< 40 μmol/L) must be managed as severe ICP. All women have accepted oral UDCA treatment. Other drugs, such as S-adenosyl methionine (SAMe) or polyunsaturated phosphatidylcholine (PPC), could also be combined with UDCA if necessary, and termination of pregnancy will not exceed 37 weeks (Fig. 1) [23].

The data collection was anonymously analysed between women conceived by ART and SC. First, we compared baseline characteristics in the two groups, including age, BMI, twin chorionicity, scarred uterus, gestational complications such as placenta previa, placental abruption, gestational diabetes mellitus (GDM), preeclampsia, premature rupture of membrane (PROM), and twin comorbidities including twin-to-twin transfusion syndrome (TTTS), selective intrauterine growth restriction (sIUGR), and twin reversed arterial perfusion sequence (TRAPS). The primary assessment is perinatal outcomes, including gestational age (GA) at delivery, cesarean delivery, birth weight, meconium-stained amniotic fluid (MSAF), Apgar score < 7 at 5 min, neonatal intensive care unit (NICU) admission, and still-birth rate. In addition, the effect of UDCA treatment is secondary assessment, so we analysed different elevated TBA levels and drug usage by subgroup analysis, and compared therapeutic outcomes based on changing trends of TBA. Liver enzymes during medication, mainly alanine aminotransferase (ALT) and aspartate aminotransferase (AST) have also has been compared.

### Statistical analyses

Statistical analysis was performed by SPSS for Windows version 22.0 (IBM, Armonk, NY, USA). Continuous variables are presented as medians (interquartile ranges) because they did not conform to a normal distribution, and categorical variables are expressed as frequency n (%). Then, Kruskal–Wallis or chi-square test was used to compare the ART and SC groups. A value of *p* < 0.05 was considered statistically significant.

## Results

From January 1^st^, 2014 to January 31^st^, 2019, the incidence of ICP was 5.85% (3380 of 57,815) in a singleton pregnancy, and 21.9% (903 of 4,132) in twin pregnancies by searching for ICD-10. After the exclusion of 39 twin pregnancies not diagnosed or untreated ICP by correction, a total of 864 twin pregnancies were included, 500 women in the ART group and 364 in the SC group. Figure 1 is presented as a detailed inclusion process.

The baseline characteristics are presented in Table 1. The median pregestational BMI [21.4 (20.0–23.4) vs 21.0 (19.5–22.8), *p* = 0.005] and ratio of advanced maternal age (26.4% vs 13.2%, *p* = 0.001) in the ART group were higher than SC group. There were more maternal complications in the ART group, including placenta previa (7.4% vs 2.7%, *p* = 0.003) and GDM (34.4% vs 23.9%, *p* = 0.001), but fewer scarred uteri (3.8% vs 14.8%, *p* = 0.001) and PROMs (13.4% vs 21.4%, *p* = 0.002) than in the SC group. There was no significant difference between the two groups, including placental abruption (2.4% vs 1.1%, *p* = 0.161), preeclampsia (65% vs 66.2%, *p* = 0.712), dichorionic twins (35% vs 31.9%, *p* = 0.803) and twin comorbidities (16.8% vs 22.0%, *p* = 0.55), such as twin-to-twin transfusion syndrome (TTTS), selective intrauterine growth restriction (sIUGR), and twin reversed arterial perfusion sequence (TRAPS).

**Table 1:** Baseline characteristics of the study groups

Characteristics	ART group (*N* = 500)	SC group (*N* = 364)	*P* value
Advanced age(≥ 35 years),n(%)	132(26.4%)	48(13.2%)	.001^a^
BMI (kg/m^2^), median(*P*_25_ ~ *P*_75_)	21.4(20.0 ~ 23.4)	21.0(19.5 ~ 22.8)	.005^a^
Dichorionic twins, n(%)	175(35.0%)	116(31.9%)	.803^a^
Scarred uterus, n(%)	19 (3.8%)	54 (14.8%)	.001^a^
Placenta previa, n(%)	37 (7.4%)	10 (2.7%)	.003^a^
Placenta abruption, n(%)	12 (2.4%)	4 (1.1%)	.161
GDM, n(%)	172 (34.4%)	87 (23.9%)	.001^a^
Preeclampsia, n(%)	325 (65%)	241(66.2%)	.712
PROM, n(%)	67 (13.4%)	78 (21.4%)	.002^a^
Twin comorbidities, n(%)	84 (16.8%)	80 (22.0%)	.055^a^

*ART* assisted reproductive technology, *SC* spontaneous conception, *BMI* body mass index, *GDM* gestational diabetes mellitus, *PROM* premature rupture of membranes, *TTTS* Twin comorbidities include twin-to-twin transfusion syndrome, *sIUGR* selective intrauterine growth restriction, *TRAPS* twin reversed arterial perfusion sequence, etc. ^a^ Denotes significant values if *p* < 0.05

Subgroup analysis displayed in Table 2, there was no statistic difference on serum TBA levels between ART and SC group, including TBA at diagnosis of ICP (*p* = 0.58), TBA peak(*p* = 0.103) and TBA at delivery(*p* = 0.211), but the median level of ALT [140.0(60.0 ~ 241.5) vs153.50(80.0 ~ 286.7), *p* = 0.021], AST [102.0(51.0 ~ 185.0) vs 118.00(63.2 ~ 193.0), *p* = 0.017] in the ART group was lower than SC group. Although more patients in the ART group accepted UDCA treatment with double combinations (82% vs 73.9%, *P* = 0.075) or triple combinations (64.1% vs 61.5%, *p* = 0.588), there were no significant differences in therapeutic outcome (inefficacy rate 47.2% vs 47.0%, improvement rate 30.4% vs 31.6%, remission rate 22.4% vs 21.4%, *p* = 0.926) between the two groups.

**Table 2:** Biochemical indicators and medication in the study groups

	ART group (*N* = 500)	SC group (*N* = 364)	*P* value
GA at diagnosis < 32 weeks,n(%)	59(16.2%)	47(9.4%)	.003^a^
TBA (umol/L) at diagnosis,n(%)			.058
≥ 10, < 40	427(85.7%)	295(81%)	
≥ 40, < 100	67 (13.5%)	62(17.0%)	
≥ 100	6 (0.8%)	7 (2.0%)	
ALT (U/L), median(*P*_25_ ~ *P*_75_)	140.0(60.0 ~ 241.5)	153.50(80.0 ~ 286.7)	.021^a^
AST (U/L), median(*P*_25_ ~ *P*_75_)	102.00(51.0 ~ 185.0)	118.00(63.2 ~ 193.0)	.017^a^
TBA Peak (umol/L), n(%)			.103
≥ 10, < 40	354(70.8%)	241(66.2%)	
≥ 40, < 100	133(26.6%)	104(28.6%)	
≥ 100	13 (2.6%)	19(5.2%)	
TBA (umol/L) at delivery, n(%)			.211
≥ 10, < 40	434(86.8%)	305(83.8%)	
≥ 40, < 100	59(11.8%)	52(14.3%)	
≥ 100	7 (1.4%)	7(1.9%)	
Medication of UDCA, n(%)			
Double-combinations	410(82%)	269(73.9%)	.075
Triple-combinations	321(64.1%)	224(61.5%)	.588
Medication time(days), median(*P*_25_ ~ *P*_75_)	17.00(7.00 ~ 31.00)	17.00(7.00 ~ 33.00)	.816
Changing trend of TBA, n(%)			.926
inefficacy	236(47.2%)	171(47.0%)	
improvement	152(30.4%)	115(31.6%)	
remission	112(22.4%)	78 (21.4%)	

*ART* assisted reproductive technology, *SC* spontaneous conception, *GA* gestational age, *TBA* total bile acid, *ALT* alanine aminotransferase, *AST* aspartate aminotransferase, *UDCA* Ursodeoxycholic Acid, Double- or triple-combinations means plus S-adenosyl-methionine (SAMe) or/and Polyene Phosphatidylcholine Capsules (PPC), ^a^ Denotes significant values if *p* < 0.05

Perinatal outcomes are shown in Table 3. Compared with the SC group, the incidence of preterm delivery was lower in the ART group (GA at delivery < 34 weeks, 13.2% vs 21.7%, GA at delivery ≥ 34, < 37 weeks,69.0% vs 68.2%, *P* = 0.000), the median birth weight in the ART group was higher [2280.0 (1950.0 ~ 2520.0) vs 2140.0 (1815.0 ~ 2420.0), *p* = 0.001], and the NICU admission rate was significantly lower (37.2% vs 52.4%, *p* = 0.001). Other neonatal outcomes, including the rate of cesarean delivery (91.4% vs 88.2%,*p* = 0.119), MSAF (12.2% vs 15.6%, *P* = 0.121), still-birth (1.4% vs 1.1%, *p* = 0.697) and Apgar score < 7 at 5 min (2.1% vs 2.0%, *p* = 0.919), were not significantly different.

**Table 3:** Main perinatal outcomes of the study groups

	ART group (*N* = 500)	SC group (*N* = 364)	*P* value
GA at delivery(weeks), n(%)			.000^a^
term, ≥ 37w	93(18.6%)	34(9.3%)	
preterm, < 34w	66(13.2%)	79(21.7%)	
preterm, ≥ 34, < 37w	341(68.2%)	251(69.0%)	
Caesarean delivery, n(%)	457(91.4%)	321(88.2%)	.119
birth weight(g), median(P25 ~ P75)			
baby 1	2350.0(2100.0 ~ 2570.0)	2210.0(1910.0 ~ 2450.0)	.001^a^
baby 2	2280.00(1950.0 ~ 2520.0)	2140.0(1815.0 ~ 2420.0)	.001^a^
MSAF,n(%)	61(12.2%)	57(15.6%)	.121
Apgar score < 7 at 5 min, n(%)	11 (2.1%)	8 (2.0%)	.919
NICU admission, n(%)	182(37.2%)	184(52.4%)	.001^a^
Still-birth, n(%)	7(1.4%)	4(1.1%)	.697

*ART* assisted reproductive technology, *SC* spontaneous conception, *GA* gestational age, *MSAF* meconium stained amniotic fluid, *NICU* neonatal intensive care unit. ^a^ Denotes significant values if *p* < 0.05

## Discussion

ICP is one of the main complications during pregnancy, and the incidence during twin pregnancies is higher than that during singleton pregnancy because of widely used ART, but there is no conclusion regarding whether ART may significantly increase adverse perinatal outcomes in ICP [6, 19, 22]. Moreover, UDCA has always been recommended for reducing bile acid, but controversy remains if it could improve perinatal outcomes in twin pregnancies with ICP [2]. Our study found that although women conceived by ART may increase the risk of early-onset ICP, it may not increase adverse perinatal outcomes in comparison with spontaneous conceptions. To evaluate the therapeutic effect of UDCA by subgroup analysis, we also found that UDCA may not improve the perinatal outcomes of twin pregnancies.

The wide application of ART has increased the risk of multiple pregnancies, and maternal complications during twin pregnancies such as preeclampsia, intrahepatic cholestasis of pregnancy, gestational diabetes, and twin comorbidities, also more frequently occur [24], so the correlation between ART and perinatal outcomes has gained much attention. When compared to SC, current studies agree that ICP develops earlier and has a higher rate in ART-conceived singleton and twin pregnancies [25, 26]. However, uncertainty remains regarding whether ART may lead to adverse pregnancy outcomes in twin pregnancies of ICP [27]. Yang finished a multicenter retrospective analysis from 14 provinces and 39 different hospitals, and a total of 112,403 deliveries of singleton and twin pregnancies were included. Their study suggested that ART pregnancy may increase preterm delivery, increase low birth weight and low 5-min Apgar, also increase the rate of neonatal death [25]. Similarly, another retrospective analysis showed that even after adjusting for early-onset ICP and chorionicity, in vitro fertilization-embryo transfer (IVF-ET) may increase premature delivery and neonatal asphyxia [28]. Our study also demonstrated that ART may increase the risk of early-onset ICP in twin pregnancies, but it seems not to increase low 5-min Apgar and MSAF, even has a lower incidence of preterm delivery, lower newborn weight, and NICU admission, which is opposite to previous investigations. A possible reason may be explained, previous studies have not evaluated drug usage and therapeutic effects concurrently, but we only included women who accepted UDCA treatment.

Current guidelines recommend that pregnant women diagnosed with severe ICP should be managed actively because TBA ≥ 40 μmol/L has an increased risk for adverse perinatal outcomes, especially stillbirth [5, 29]. For twin pregnancies, women with TBA < 40 μmol/L should also be diagnosed with severe ICP according to Chinese guidelines, so most of the women in our study had mildly elevated TBA but were regarded as severe ICP. However, Cui found that women with bile acids greater than 40 μmol/L have no increased incidence of stillbirth but increased iatrogenic preterm delivery [5]. Ovadia performed meta-analyses to identify that the risk of stillbirth is significantly increased when women have serum bile acids of 100 μmol/L or more, but they both concluded in singleton pregnancy [7]. The corrected stillbirth rate in our study was approximately 1.2% (11/864), excluding known reasons such as fetal malformation, twin-to-twin transfusion syndrome (TTTS), and abnormal umbilical cord. Among those stillbirths, only half of them had serum bile acids beyond 40 μmol/L. Moreover, the present study showed that the rate of preterm delivery between 34 ~ 37 gestational weeks was almost 68% ~ 69%, which may result from active termination of twin pregnancies despite an elevated TBA level of less than 40 umol/L. These conclusions mean that active management by obstetricians may contribute to decreasing stillbirth in severe ICP, but the prevalence of iatrogenic preterm delivery also needs to be given more attention.

Pharmacologic treatment for ICP aims to reduce maternal pruritus or adverse perinatal outcomes, and some meta-analyses have reported UDCA benefits in relieving pruritus and improving laboratory abnormalities, Currently, it is the first-line agent for ICP and without known adverse effects on the fetus [30, 31]. S-adenosyl-methionine (SAMe) and cholestyramine can also be considered alternative drugs for patients who cannot take UDCA or have continued symptoms on the maximum dosage of UDCA. Another Chinese drug named polyene phosphatidylcholine capsules (PPCs) has also been reported to reduce liver enzymes. Despite aggressive medication for ICP, it does not seem to improve pregnancy outcomes. A meta-analysis of thirteen randomized controlled trials (RCTs) found that neither UDCA alone nor a drug combination could significantly improve pruritus and perinatal outcomes in both singleton and twin patients [15]. Another multicenter prospective RCT found that compared with placebo, UDCA could not reduce the incidence of stillbirth, preterm delivery, neonatal hospitalization, and other adverse outcomes in patients with ICP, suggesting that the routine usage of UDCA for women with mild ICP should be reassessed [13]. The latest meta-analysis including a total of 34 studies by Ovadia and colleagues shows that the stillbirth rate is approximately 0.7% in ICP patients who underwent UDCA, concluding that UDCA has no significant effect on the prevalence of stillbirth, but it has the opposite result of a lower still-birth rate (0.2%) when considering only randomized controlled trials [14]. Our study only included women who had taken UDCA when diagnosed with ICP, almost 82% of them plus taking SAMe, and 64% triply using cholestyramine, but subgroup analysis showed no differences in serum TBA levels and stillbirth rate.

Our study has some strengths. It included a large sample size of twin pregnancies with ICP. All women included accepted medication, and the drug usage and serum TBA levels during medication were also compared. In addition, every case of data collection from medical records was redefined by diagnostic criteria. This study still has some limitations. First, it is a retrospective analysis that may be prone to selection bias. Second, a higher rate of placenta previa and PROM appears in ART conceived women, which may affect pregnancy outcomes. Third, even though preterm delivery was compared as the main result, iatrogenic preterm birth was not been analysed separately. Fourth, neonatal long-term outcomes were not assessed.

## Conclusion

In conclusion, ART-conceived twin pregnancies have a higher risk of early-onset ICP, but it seems to have no association with adverse perinatal outcomes. For twin pregnancies complicated with elevated bile acids, the UDCA used for improving perinatal outcomes was limited. Our conclusions still need more prospective randomized controlled studies to confirm.

## Data Availability

The data of study are not publicly available due to ethical and legal restrictions. However, upon request, data may be available from Institutional Review Board of West China Second Hospital of Sichuan University.

## Abbreviations

ICP: Intrahepatic cholestasis of pregnancy
ART: Assisted reproductive technology
SC: Spontaneous conception
TBA: Total bile acid
ALT: Alanine aminotransferase
AST: Aspartate aminotransferase
UDCA: Ursodeoxycholic acid
MSAF: Meconium stained amniotic fluid
NICU: Neonatal intensive care unit
